# Association between rural clinical clerkship and medical students’ intentions to choose rural medical work after graduation: A cross-sectional study in western China

**DOI:** 10.1371/journal.pone.0195266

**Published:** 2018-04-02

**Authors:** Jinlin Liu, Bin Zhu, Ying Mao

**Affiliations:** 1 School of Public Policy and Administration, Xi’an Jiaotong University, Xi’an, Shaanxi, China; 2 Department of Public Policy, City University of Hong Kong, Hong Kong, China; Rural Clinical School of Western Australia, AUSTRALIA

## Abstract

**Background:**

A large number of programs have been implemented in many countries to increase the healthcare workforce recruitment in rural and remote areas. Rural early exposure programs for medical students have been shown to be effective strategies. However, no related studies have been reported before in China. This study was carried out to determine the association between medical students’ participation in rural clinical clerkships and their intentions to choose rural medical work after graduation from western medical schools in China.

**Methods:**

Based on a two-stage random sampling method, the cross-sectional survey was carried out in ten western provinces in China. A brief questionnaire filled in by medical students was used for data collection. A total of 4278 medical students participated in the study. The response rate was approximately 90.34%. Pearson’s chi-squared tests and binary logistic regression analyses were performed for data analyses.

**Results:**

Approximately 52.0% of medical students disclosed intentions to work in rural medical institutions after graduation. Only one in five participants had experience with a rural clinical clerkship. Rural clinical clerkships were significantly associated with medical students’ intentions to work in rural medical institutions (OR: 1.24, 95%CI: 1.05–1.46); further analyses indicated that such clerkships only had a significant impact among the medical students with an urban background (OR: 2.10, 95%CI: 1.48–2.97). In terms of the sociodemographic characteristics, younger age, low level of parental education, majoring in general practice, and studying in low-level medical schools increased the odds of having intentions to engage in rural medical work among medical students; however, rural origins was the only positive univariate predictor. In addition, the predictors of intentions to choose rural medical work were different between medical students with a rural background and those with an urban background.

**Conclusions:**

Rural clinical clerkship is likely to increase the odds of having intentions to work in rural medical institutions after graduation among medical students in western China, especially for those with an urban background. Related policy makers could consider developing compulsory rural clerkship programs and implement them among medical students to increase early rural exposure.

## Introduction

No healthcare without a workforce has been a universal truth [1]. However, shortages and misdistribution of the qualified healthcare workforce have become global concerns affecting nearly all countries, especially less-developed countries. Meanwhile, the challenges of the healthcare workforce are most severe in rural and remote areas in these countries. In China, while 43.9% of the population live in rural areas [2], only 37.7% of healthcare providers practice there [3]. How to attract additional healthcare workers to work in rural and remote areas has drawn considerable attention.

Medical education plays a vital role in cultivating medical students and supplying future healthcare providers. However, despite an increasing number of students graduating from medical schools globally, a shortage of rural healthcare providers persists. A recent systematic review revealed that medical students’ career intentions to work in rural locations were low (10%-20%) in some low- and middle-income countries [4], such as Nepal [5], Bangladesh [6], sub-Saharan Africa [7], Ethiopia [8], South Africa [9], and India [10]. In addition, a survey of five countries in Asia reported approximately 60% of medical students in Bangladesh and Thailand had positive attitudes towards working in rural areas, followed by 50% in China and India, and 33% in Vietnam [11]. Furthermore, many determinants contribute to medical students’ career intentions and decision to work in rural locations. It is important and difficult for medical students to make the final decision on where to start their career, which will influence their future career opportunities and development, as well as their family and social life. Puddey et al. found that origin, age, gender, registration type, and school type were significantly associated with rural practice among medical students [12]. Budhathoki et al. developed a conceptual framework for medical students’ motivations to work in rural areas following graduation, including health facilities, personal lifestyle, medical training and curriculum, medical school, and policy-related factors [4]. A rural background (i.e., being brought up in a rural area), training in rural areas with a community-based curriculum, early exposure to the community during medical training, and rural location of the medical school motivated medical students to work in rural areas [4,13]. In addition, gender, personal job concerns, and family factors were identified as associated with medical students’ intentions to engage in rural medical work [11,14]. Bowman et al. considered that one significant explanation for why so few medical graduates were willing to work in rural areas was lack of contact with rural communities, people, and practitioners [15], which marked the importance of early rural exposure before graduation of medical students.

In 2010, the World Health Organization (WHO) proposed a series of evidence-based global policy recommendations [16] in response to the global challenge of an insufficient health workforce in rural and remote areas. Among these recommendations, one was to expose medical students to rural community experiences and clinical rotations, as these had a positive influence on attracting and recruiting more medical students to work in rural areas after graduation [17]. A large number of medical schools in many countries had implemented the relevant strategy of offering medical students opportunities to practice in rural areas as part of their educational experience. In Japan, the University of Tsukuba created a one-day early-exposure program to provide fresh students with experience in rural practices, which demonstrated a positive impact on medical students’ interest in rural medical practice, with an increase from 39.0% (pre-program) to 61.0% (post-program) [18]. In Australia, medical students from three universities (the Universities of Sydney, Wollongong and Adelaide) were involved in a longitudinally integrated placement program that enabled medical students to live and work in a rural and remote setting and surrounding regions for 6–12 months for clinical learning, and this powerfully influenced their intentions to practice rurally [19]. In America, a larger proportion of medical graduates enrolled in the Rural Physician Associate Program (RPAP) at the University of Minnesota Medical School [20], the Rural Medical Education Program (RMED) at State University of New York Upstate Medical University [21], and the Physician Shortage Area Program (PSAP) at Jefferson Medical College [22] worked in rural areas than those who did not participate in these programs. In New Zealand, a compulsory and week-long rural externship program at Otago University’s School of Pharmacy increased the interest and enthusiasm (48% pre- versus 73% post-externship) for rural work among pharmacy undergraduates [23]. Many other studies on medical students also demonstrated the positive effect of rural rotations or training on willingness to work rurally [24–27].

In China, a number of programs have been developed over the past few decades to increase the recruitment and retention of rural primary health care providers. The best known among these is the “Rural-oriented Tuition-waived Medical Education (RTME),” an ongoing education program that only recruits medical students with a rural background and provides scholarships in return for obligated medical service in the rural township hospitals of western and middle regions in China. However, no relevant programs on early rural exposure (i.e., rural clinical clerkship) for medical students before graduation have been developed and reported before, and no related studies have been found in China.

Based on a cross-sectional survey with a large sample size of medical students in western medical schools of China, this study aimed to explore: 1) medical students’ intentions of rural medical work after graduation; 2) the rural clinical clerkship situation; 3) association between rural clinical clerkships and rural working intentions; and 4) other determinants of rural working intentions.

## Methods

### Study design and participants

The cross-sectional study was a part of the collaborative research project, “*Situational Analysis and Policy Evaluation of Development and Retention of Human Resources for Health in Rural Western China*,” funded by the China Medical Board (CMB) and technically supported by the WHO.

The survey was carried out in ten western provinces in China (i.e., Gansu, Kweichow, Inner Mongolia, Ningxia, Qinghai, Shaanxi, Sichuan, Tibet, Xinjiang, and Yunnan). A total of ten research teams from the ten provinces participated in the survey coordinated by Xi’an Jiaotong University.

A two-stage random sampling method was used. The sample sizes for medical schools and medical students were negotiated and determined by all the co-PIs of the CMB-funded project and experts from the WHO under consideration of the survey duration and budget. First, in every province, no more than six medical schools were selected randomly, including one or two technical secondary medical schools (if available), one or two junior medical colleges (if available), and one or two medical universities (if available). Second, no more than 100 medical students in each medical school (third-, fourth-, or fifth-year grade) were selected randomly. Approximately a total of 5000 medical students were randomly selected; however, only those medical students who were willing to participate in the survey answered the questionnaires. Lastly, 4517 questionnaires were collected. The response rate was approximately 90.3%. Of which, 94.7% (4278) were retained in the study after the data were incorporated and checked by Xi’an Jiaotong University. Two hundred and thirty-nine questionnaires were excluded because of missing values regarding intentions toward rural medical work after graduation.

### Data collection and variable measurement

A brief, structured questionnaire was developed for data collection. The completed questionnaire was developed initially by the research team of Xi’an Jiaotong University according to the research objectives of the CMB-funded project. Then, each of the other nine research teams from nine western provinces in China validated the questionnaire by group discussion. Some research teams made small-scale pre-surveys on medical students to validate the logic and rationality of the questionnaire. Finally, all ten research teams revised and completed the questionnaire together. During this process, Dr. Fethiye Gulin Gedikg and Dr. Chunmei Wen, two experts from the WHO, provided sufficient technical support for the questionnaire design. Data were collected from June to December 2013. All the questionnaires were completed by the medical students themselves. Considering the objectives of this study, we extracted only the relevant variables and data. They consisted of three sections, including general sociodemographic information, clinical clerkship, and intention to engage in rural medical work after graduation.

Variables used to measure sociodemographic characteristics were as follows: (1) gender: female and male; (2) age was a continuous variable and was divided into two groups: ≤ 20 years and ≥ 21 years; (3) residence: rural and urban; (4) monthly per capita income of the student’s family: low (< 1000 Yuan), medium (1000–4999 Yuan), and high (≥ 5000 Yuan); (5) education level of the student’s parents: low (≤ primary school), medium (junior high school), and high (≥ senior high school); (6) grade: third year, fourth year, and fifth year; (7) specialty: general practice, clinical medicine, public health, and other; (8) type of medical school: technical secondary, junior college, and university (undergraduate).

Rural clinical clerkship was a binary variable (i.e., yes or no), determined using two questions, “*do you have experience of a clinical clerkship*? *(yes or no)*” and “*if yes*, *where do you practice*? *(urban*, *rural or other)*.” Based on these questions, medical students who had experience with rural clinical clerkships meant that they had experience of a clinical clerkship in rural medical institutions before this survey, and medical students who had no experience of rural clinical clerkships consisted of two groups: those who had no experience of a clinical clerkship and those who had experience of a clinical clerkship in urban or other medical institutions. In addition, since we did not systematically collect data on the duration of clinical clerkship, we could not further analyze its association with medical students’ intentions toward rural medical work after graduation.

Intention to engage in rural medical work was determined using a dichotomous question, “*Are you willing to work in rural medical institutions after graduation*?*”* answered with either yes or no.

### Statistical methods

Continuous variables (i.e., age) were tested for normality first with a 1-Sample K-S test. These variables presented an abnormal distribution and were described using the “median” and “interquartile range (IQR).” Categorical variables were described by “number” and “percentage.”

A Pearson’s chi-squared test was used to assess the differences in the proportions of sociodemographic characteristics and clinical clerkship between the medical students with and without intentions of rural medical work. Chi-squared values and *P*-values were reported.

A binary logistic regression analysis was performed to determine the association between rural clinical clerkships of medical students and their intentions to carry out rural medical work after graduation, and we identified additional influencing factors. Intention to engage in rural medical work was set as the dependent variable, rural clinical clerkship was set as the independent variable, and the nine sociodemographic characteristics were set as the controlled variables. Crude odds ratio (OR) with 95% confidence interval (CI) was estimated for the independent variable and each controlled variable first. When conducting the multivariate binary logistic regressions, the controlled variables that were significant in univariate analyses were introduced into the regression model to further analyze the association between the independent variable and the dependent variable. In addition, we further explored the association between rural clinical clerkships and intentions to engage in rural medical work after graduation among medical students coming from rural and urban areas separately and compared their differences. The results were presented as the adjusted OR with 95%CI. All the significance levels were set at *P*-value < 0.05.

The Statistical Package for Social Sciences 24.0 (SPSS, IBM, Armonk, New York, USA) for MAC was used for data analysis.

### Ethics

This study was approved by the Ethics Committee of the School of Medicine of Xi’an Jiaotong University (China), and the approval number was 2014189. The questionnaire was anonymous and verbal informed consent was obtained from all participating medical students.

## Results

### Sociodemographic characteristics

A total of 4278 medical students participated in the study. Sociodemographic characteristics are summarized in Table 1. A total of 67.5% of the respondents were female. The median age was 21 years (IQR: 20–22 years), and 69.7% were older than 21 years. More than two thirds (68.6%) were from rural areas. Approximately 48.9% came from low-income families (family per capita income was lower than 1000 Yuan per month). 69.0% of medical students’ fathers attained medium- or low-level education (i.e., primary school and junior high school), and more than half (51.3%) of their mothers only attained a low-level education. Most of the medical students were in the fourth-year (56.5%) and fifth-year (33.5%). Approximately seven in ten majored in clinical medicine, followed by general practice (15.1%) and public health (13.0%). Moreover, three in five were studying in the universities, followed by junior medical colleges (27.7%) and technical secondary medical schools (11.5%).

**Table 1:** Sociodemographic characteristics of medical students.

Characteristics	N (%)	Intention to choose rural medical work		Chi-squared test	
		Yes, n (%)	No, n (%)	Value	*P*-value
**Gender (n = 4278)**				1.345	0.253
Female	2889 (67.5)	1521 (52.6)	1368 (47.4)		
Male	1389 (32.5)	705 (50.8)	684 (49.2)		
**Age (years, n = 4278)**				37.623	**0.000**
≤ 20	1297 (30.3)	767 (59.1)	530 (40.9)		
≥ 21	2981 (69.7)	1459 (48.9)	1522 (51.1)		
**Residence (n = 4277)**				71.438	**0.000**
Rural	2935 (68.6)	1655 (56.4)	1280 (43.6)		
Urban	1342 (31.4)	570 (42.5)	772 (57.5)		
**Income (monthly per capita income of student’s family, Yuan, n = 4255)**				30.847	**0.000**
< 1000	2079 (48.9)	1148 (55.2)	931 (44.8)		
1000–4999	1816 (42.7)	929 (51.2)	887 (48.8)		
≥ 5000	360 (8.5)	143 (39.7)	217 (60.3)		
**Education of student’s father (n = 4271)**				89.006	**0.000**
Low	1424 (33.3)	859 (60.3)	565 (39.7)		
Medium	1525 (35.7)	803 (52.7)	722 (47.3)		
High	1322 (31.0)	560 (42.4)	762 (57.6)		
**Education of student’s mother (n = 4270)**				95.428	**0.000**
Low	2190 (51.3)	1287 (58.8)	903 (41.2)		
Medium	1189 (27.8)	575 (48.4)	614 (51.6)		
High	891 (20.9)	359 (40.3)	532 (59.7)		
**Grade (n = 4023)**				33.893	**0.000**
Third year	400 (9.9)	257 (64.3)	143 (35.8)		
Fourth year	2275 (56.5)	1119 (49.2)	1156 (50.8)		
Fifth year	1348 (33.5)	732 (54.3)	616 (45.7)		
**Specialty (n = 4120)**				54.633	**0.000**
General practice	622 (15.1)	361 (58.0)	261 (42.0)		
Clinical medicine	2844 (69.0)	1514 (53.2)	1330 (46.8)		
Public health	535 (13.0)	202 (37.8)	333 (62.2)		
Other	119 (2.9)	65 (54.6)	54 (45.4)		
**Type of medical school (n = 4278)**				240.215	**0.000**
Technical secondary	493 (11.5)	317 (64.3)	176 (35.7)		
Junior college	1184 (27.7)	802 (67.7)	382 (32.3)		
University	2601 (60.8)	1107 (42.6)	1494 (57.4)		

### Rural clinical clerkship

Table 2 shows the clinical clerkship experience of the medical students. Sixty-one percent of the 4278 medical students had experience of a clinical clerkship. Among these students, 2502 reported participation in clinical clerkship placements. Specifically, 880 medical students had an experience with a rural clinical clerkship and 1571 practiced in urban medical institutions.

**Table 2:** Clinical clerkship situation of medical students.

Characteristics	N (%)	Intention to choose rural medical work		Chi-squared test	
		Yes, n (%)	No, n (%)	Value	*P*-value
**Clinical clerkship (n = 4224)**				15.891	**0.000**
No	1646 (39.0)	793 (48.2)	853 (51.8)		
Yes	2578 (61.0)	1404 (54.5)	1174 (45.5)		
**Clinical clerkship placement (n = 2502)**				25.911	**0.000**
Urban	1571 (62.8)	800 (50.9)	771 (49.1)		
Rural	880 (35.2)	542 (61.6)	338 (38.4)		
Other	51 (2.0)	28 (54.9)	23 (45.1)		

### Medical students’ intentions to choose rural medical work

Fifty-two percent (2226/4278) of medical students disclosed intentions to carry out rural medical work after graduation. Table 1 shows that significant differences were observed between medical students with and without intentions to engage in rural medical work with respect to age, residence, income, education of their parents, grade, specialty, and type of medical school. Medical students with intentions to carry out rural medical work after graduation constituted higher percentages in the groups of ≤ 20 years, rural residents, low-income family, low-level education of parents, third-year grade and fifth-year grade, majoring in clinical medicine and general practice, and studying in junior colleges and technical secondary schools than those without intentions. However, no significant difference was found in terms of gender.

As shown in Table 2, significant differences were observed between medical students with and without intentions to engage in rural medical work with respect to clinical clerkship experience and clinical clerkship placement. Medical students with such intentions presented in higher percentages in the groups that had experience with a clinical clerkship and rural clinical clerkship.

Table 3 presents the crude and adjusted OR with 95%CI for each variable. Estimation of the crude OR indicated that rural clinical clerkship, age, residence, income, education of parents, grade, specialty, and type of medical school were significantly associated with medical students’ intentions to engage in rural medical work. Furthermore, Model 1 introduced eight controlled variables that were significant in previous univariate analyses, except for gender. After adjusting for these variables, the association between rural clinical clerkship and medical students’ intentions to carry out rural medical work was still significant. The medical students who had experience with a rural clinical clerkship were 1.24 times more likely to have intentions of engaging in rural medical work than those who had no related experience. Meanwhile, the medical students who were more likely to have intentions of choosing rural medical work after graduation included those who were 20 years and below, those who studied in technical secondary schools and junior colleges, and those whose parents were less educated. Meanwhile, compared with medical students who majored in general practice, those who majored in clinical medicine and public health were less likely to have intentions of engaging in rural medical work.

**Table 3:** Binary logistic regression on medical students’ intentions to choose rural medical work after graduation.

Variable	Univariate logistic regressions		Multivariate logistic regression (Model 1)	
	OR_cru._ (95%CI)	*P*-value	OR_adj._ (95%CI)	*P*-value
**Rural clinical clerkship**				
No	1		1	
Yes	**1.63 (1.40–1.90)**	**0.000**	**1.24 (1.05–1.46)**	**0.013**
**Gender**				
Female	1.08 (0.95–1.23)	0.246		
Male	1			
**Age**				
≤ 20	**1.51 (1.32–1.72)**	**0.000**	**1.23 (1.03–1.48)**	**0.027**
≥ 21	1		1	
**Residence**				
Urban	1		1	
Rural	**1.75 (1.54–2.00)**	**0.000**	1.12 (0.95, 1.32)	0.193
**Income**				
< 1000	**1.87 (1.49–2.35)**	**0.000**	1.13 (0.87–1.47)	0.377
1000–4999	**1.59 (1.26–2.00)**	**0.000**	1.22 (0.95–1.56)	0.127
≥ 5000	1		1	
**Education of student’s father**				
Low	**2.07 (1.78–2.41)**	**0.000**	**1.46 (1.20–1.78)**	**0.000**
Medium	**1.51 (1.31–1.76)**	**0.000**	**1.23 (1.03–1.48)**	**0.022**
High	1		1	
**Education of student’s mother**				
Low	**2.11 (1.80–2.48)**	**0.000**	**1.28 (1.03–1.58)**	**0.026**
Medium	**1.39 (1.16–1.65)**	**0.000**	0.93 (0.75–1.15)	0.506
High	1		1	
**Grade**				
Third year	**1.51 (1.20–1.91)**	**0.000**	0.98 (0.74–1.30)	0.896
Fourth year	**0.82 (0.71–0.93)**	**0.003**	1.02 (0.87–1.19)	0.804
Fifth year	1		1	
**Specialty**				
General practice	1		1	
Clinical medicine	**0.82 (0.69–0.98)**	**0.030**	**0.76 (0.61–0.94)**	**0.010**
Public health	**0.44 (0.35–0.56)**	**0.000**	**0.62 (0.47–0.81)**	**0.000**
Other	0.87 (0.59–1.29)	0.490	0.69 (0.44–1.07)	0.100
**Type of medical school**				
Technical secondary	**2.43 (1.99–2.97)**	**0.000**	**1.68 (1.24–2.26)**	**0.001**
Junior college	**2.83 (2.45–3.27)**	**0.000**	**2.38 (1.98–2.86)**	**0.000**
University	1		1	

Moreover, Table 4 presents the associations of rural clinical clerkships and other controlled variables with the intention to engage in rural medical work of medical students coming from rural and urban areas separately. Regarding medical students who were rural residents, estimation of the crude OR indicated that the association between rural clinical clerkships and medical students’ intentions to choose rural medical work was significant; however, when introducing seven controlled variables in Model 2, the association became insignificant. Meanwhile, three variables, namely, education of the father, specialty, and type of medical school, were statistically significant. The rural medical students who were more likely to have intentions to engage in rural medical work after graduation were those who studied in technical secondary schools and junior colleges, and those whose father had a low-level education. Meanwhile, compared with rural medical students who majored in general practice, those who majored in clinical medicine were less likely to have intentions to choose rural medical work.

**Table 4:** Binary logistic regression on intention to choose rural medical work among medical students with rural versus urban background.

Variable	Student with a rural background		Student with an urban background	
	Uni. *	Multi. ^#^ (Model 2)	Uni. *	Multi. ^#^ (Model 3)
	OR_cru._ (95%CI)	OR_adj._ (95%CI)	OR_cru._ (95%CI)	OR_adj._ (95%CI)
**Rural clinical clerkship**				
No	1	1	1	1
Yes	**1.40 (1.17–1.67)**	1.07 (0.88–1.30)	**2.04 (1.49–2.79)**	**2.10 (1.48–2.97)**
**Gender**				
Female	**1.28 (1.09–1.50)**	1.13 (0.95–1.45)	1	1
Male	1	1	**1.39 (1.11–1.74)**	**1.54 (1.20–1.99)**
**Age**				
≤ 20	**1.47 (1.26–1.72)**	1.16 (0.93–1.45)	**1.44 (1.12–1.84)**	**1.49 (1.06–2.09)**
≥ 21	1	1	1	1
**Income**				
< 1000	1.31 (0.91–1.88)		**1.74 (1.23–2.46)**	1.15 (0.77–1.71)
1000–4999	1.33 (0.92–1.92)		**1.46 (1.08–1.98)**	1.12 (0.80–1.57)
≥ 5000	1		1	1
**Education of student’s father**				
Low	**1.57 (1.29–1.92)**	**1.33 (1.06–1.68)**	**2.24 (1.65–3.03)**	**1.73 (1.17–2.56)**
Medium	1.20 (0.98–1.46)	1.15 (0.92–1.44)	**1.57 (1.22–2.01)**	1.27 (0.93–1.73)
High	1	1	1	1
**Education of student’s mother**				
Low	**1.52 (1.17–1.96)**	1.10 (0.82–1.48)	**2.04 (1.56–2.66)**	**1.48 (1.04–2.10)**
Medium	1.07 (0.81–1.41)	0.84 (0.61–1.14)	1.28 (0.99–1.67)	0.93 (0.67–1.27)
High	1	1	1	1
**Grade**				
Third year	**1.36 (1.04–1.76)**	0.94 (0.69–1.29)	**1.71 (1.03–2.81)**	0.93 (0.48–1.82)
Fourth year	**0.80 (0.68–0.94)**	1.07 (0.88–1.29)	0.91 (0.72–1.17)	0.88 (0.67–1.16)
Fifth year	1	1	1	1
**Specialty**				
General practice	1	1	1	1
Clinical medicine	0.90 (0.73–1.10)	**0.77 (0.61–0.99)**	0.74 (0.52–1.06)	0.68 (0.45–1.03)
Public health	**0.56 (0.41–0.74)**	0.79 (0.57–1.10)	**0.38 (0.24–0.58)**	**0.44 (0.27–0.72)**
Other	0.78 (0.48–1.25)	**0.57 (0.34–0.98)**	1.22 (0.60–2.50)	0.88 (0.37–2.05)
**Type of medical school**				
Technical secondary	**2.40 (1.90–3.03)**	**1.96 (1.40–2.76)**	**1.99 (1.32–3.01)**	1.45 (0.75–2.80)
Junior college	**2.74 (2.32–3.25)**	**2.77 (2.24–3.42)**	**2.25 (1.64–3.09)**	**1.79 (1.22–2.62)**
University	1	1	1	1

* Uni. = univariate regression analyses;

^**#**^ Multi. = multivariate regression analysis.

With respect to urban medical students, the association between rural clinical clerkships and medical students’ intentions to engage in rural medical work after graduation was significant when estimating the crude OR and introducing eight controlled variables in Model 3. After controlling for these variables, the urban medical students who had experience with a rural clinical clerkship were 2.10 times more likely to have intentions of rural medical work than those who had no related experience. Meanwhile, the urban medical students who were more likely to have intentions to engage in rural medical work included those who were male, those who were younger than 20 years, those who studied in junior colleges, and those whose parents received low-level education. Meanwhile, compared with urban medical students who majored in general practice, those who majored in public health were less likely to have such intentions.

## Discussion

To our knowledge, this study is the first to determine the association between rural clinical clerkships and medical students’ intentions to choose rural medical work after graduation in China. A large sample of 4278 medical students from western medical schools in China participated in the study. It provided relevant evidence from western China and adds to the growing body of literature on medical students’ perceptions of rural practice and career intentions.

The results indicated that among 4278 medical students, 52.0% had intentions to work in rural medical institutions after graduation. This percentage was higher than those found in previous studies in China. Qing et al. reported that 1523 of 4669 (32.62%) medical students from five medical universities in Guangxi, a western province of China, had a positive attitude toward working in township health centers, followed by 55.13% with a neutral attitude [14]. Another study by Zhang et al. showed that 19.1% of 2714 Chinese medical students from three medical schools from three provinces in eastern, central, and western regions of China expressed definite willingness toward a primary care career in community health centers [28]. However, the intention cannot correspond exactly to the future career choice and actual behavior. Playford et al. found that the link between medical students’ intentions to work rurally and their actual behavior after graduation was tenuous [13]. Meanwhile, in the present study, only those students who were willing to participate in the survey were invited to fill in the questionnaire, which might have introduced a positive selection bias toward medical students with a prior preference toward rural medical work in this study. Based on the above and given China’s dearth of highly trained health workers in rural areas, an intention of more than 50% can hardly be translated into actual rural practice of medical students, which should be a focus of attention.

Our study reported that only 20.5% of medical students had experience with a rural clinical clerkship, and approximately 40% did not have any experience with a clinical clerkship, which might be because there were no relevant compulsory programs in medical schools for students to participate in the clinical clerkship let alone participate in the rural clinical clerkship. Additionally, some students who had intentions of engaging in rural medical work might not actively seek opportunities to participate in rural clinical clerkships by themselves. It seemed likely that most of the medical students who chose rural medical institutions for clinical clerkships were interested in rural medical work. Consistent with previous studies conducted in Japan [18], Australia [19], America [20–22], and New Zealand [23], this study demonstrated a significant association between medical students’ experiences with rural medical clerkships and their intentions to choose rural medical work after graduation. The results indicated that medical students who had experience with a rural clinical clerkship were 1.25 times more likely to have intentions to work in rural medical institutions after graduation than those without relevant experience. However, a contradictory case was reported in Botswana, indicating that rural exposure during training was not enough to entice medical students to practice in rural areas [29]. Early rural exposure, such as a clinical clerkship, rotation, and training, was beneficial for medical students to learn more about rural communities, people, and health issues and, thus, to improve their interest in rural medical work [30]. Meanwhile, the experience showed them that rural practice was not as “bad” as they had previously thought [29]. In addition, our study further demonstrated that the association between rural clinical clerkships and intentions to engage in rural medical work was different between medical students with a rural background and those with an urban background. Urban medical students with experience of a rural clinical clerkship were 2.10 times more likely to have an intention to work in rural areas than those without related experience; however, it was not significantly associated with the rural medical students’ intentions. This might be attributed to medical students with a rural background already understanding rural health issues better than urban students, resulting in more intentions even without the experience of a rural clinical clerkship. However, this was inconsistent with a prior study conducted in Australia by Playford et al. [31], in which the experience of early rural exposure was a significant additive to the rural background that had an impact on medical students’ rural practice after graduation.

The present study showed that residence was not significant when adjusting for other variables. However, the results of a univariate analysis manifested that a rural background of residence was significantly associated with medical students’ intentions to work in rural medical institutions after graduation. In this study, 56.4% of rural medical students had intentions to engage in rural medical work, which was significantly higher than that (42.5%) among urban medical students; univariate regression showed that medical students with a rural background were 1.75 times more likely to have an intention to choose rural medical work than those with an urban background. Residence was a very important variable found in the published evidence from Botswana [29], Nepal [32], and Ethiopia [8]. A prior systematic review has stated that a rural background appeared to be the single factor most strongly associated with rural practice of medical students [33]. Playford et al. reported that medical graduates from a rural background were almost four times as likely as urban origin graduates to practice rurally [13]. Compared to those with an urban background, the medical students with a rural background were more familiar with rural conditions. Additionally, a greater understanding, especially about the poor rural health system, motivated them to go back and help rural areas after graduation. Some even considered rural service a part of their duty as a rural citizen [34]. This was also the basis for a large number of education policies or projects in many countries with the purpose of improving the healthcare workforce shortage in rural areas that were implemented by recruiting medical students with a rural background, for example, the RTME policy in China. In South Africa, Australia, Canada, and America, recruiting rural origin scholars who would return to work in rural areas after training has been shown to be an effective strategy for increasing staffing levels at rural and remote facilities [35–37].

In addition to rural clinical internships, this study identified four influencing factors associated with medical students’ intentions to choose rural medical work. The first factor was age. Medical students who were younger than 20 years were 1.23 times more likely to have intentions to engage in rural medical work than those aged older than 21 years. Increased age significantly decreased their intentions. For the older medical students who were about to graduate, they had been facing career choices, which could have been affected by many factors. Their intentions would be closer to actual career choices. However, this was inconsistent with the results of previous studies. Borracci et al. found that older age of Argentine medical students was highly associated with their willingness to practice medicine in rural or underprivileged areas [24]. Puddey et al. [12] and Playford et al. [31] reported that older age was a significant factor for working in rural areas or in areas of low socio-economic status among medical graduates in Australia.

The second factor was the education of parents. Medical students whose parents were less educated were more likely to have intentions of choosing rural medical work than those whose parents received high-level education. However, a survey among medical students from five countries in Asia by Chuenkongkaew et al. reported a contradictory finding that parents’ education was not significantly associated with their attitudes towards working in rural areas [11].

The specialty of medical students was the third factor. Medical students who majored in general practice seemed to be more likely to have rural working intentions than those majoring in clinical medicine or public health. This was concordant with Puddey et al.’s study, which indicated that having a general practice qualification was significantly associated with ultimately practicing in areas of low socio-economic status among medical graduates in Australia [12]. In China, there is a shortage of general practitioners in primary healthcare institutions. It seems likely that to recruit and train more general practice medical students would be very beneficial.

The fourth factor was the type of medical school. Compared with medical students studying in universities, those studying in technical secondary schools and junior colleges were 1.68 and 2.38 times more likely to have intentions to work in rural areas after graduation, respectively. Medical students in China can obtain their medical degrees through different educational programs. A degree from a technical secondary school generally equates to a degree from a senior high school; junior college provides a 3-year training program for a diploma certificate; and universities provide 5-, 7-, and 8-year programs for bachelor’s, master’s and doctor’s degrees, respectively [28]. Medical graduates need to attend the medical practitioner examination in China, and once they pass the examination, they have the independent prescribing right. Medical graduates in universities (bachelor’s degree) can attend the examination after one year’s internship under the direction of medical practitioners. However, medical graduates in technical secondary schools and junior medical colleges need to pass the assistant medical practitioner examination after one year’s internship first. After that, they can attend the medical practitioner examination after two years’ (graduates in junior medical colleges) and five years’ (graduates in technical secondary schools) internship under the direction of medical practitioners [38]. A similar finding in China was reported by Qing et al. in that a three-year program (junior college) could promote the medical students’ intentions to work in a rural area [14]. It was understandable that most of the medical students who were studying in universities and spending more time, money, and energy for the higher degree intended to work in urban high-level hospitals, where working and living conditions were better than those in rural areas.

This study demonstrated differences in influencing factors related to intentions to choose rural medical work after graduation between the medical students with a rural background and those with an urban background. In addition to rural clinical clerkships mentioned before, gender was a factor influencing the intention to choose rural medical work among medical students with an urban background. Male students were 1.54 times more likely than female students to have such intentions. However, this effect of gender is controversial. Qing et al. [14], Huntington et al.[5], and Van Wyk et al. [39] reported that male students were more likely than female students to practice in rural areas in China, Nepal, and South Africa, respectively. However, Chuenkongkaew et al. [11] found that male students were less positive about rural medical work than female students; Borracci et al. [24], Zhang et al. [28] and Puddey et al. [12] demonstrated an insignificant association between the gender of medical students and their intentions. In addition, age and mother’s education were influencing factors of intentions of medical students with an urban background, whereas they were insignificant for rural medical students. The student’s father’s education, specialty, and type of medical school were significantly associated with the intention to choose rural medical work in medical students with either an urban background or a rural background.

This study has several implications. It highlighted the importance of rural clinical clerkships. Therefore, related policy makers, medical schools, and development agencies in China might wish to develop compulsory rural clinical clerkship programs for medical students, especially for those with an urban background, to improve their understanding of and interest in rural life and medical work. Meanwhile, the importance of rural origins could not be neglected. Therefore, in terms of the ongoing education program in China; i.e., RTME, it can further expand its recruitment of medical students with a rural background. In addition, considering that medical students who were majoring in general practice and studying in junior medical colleges may be more willing to work in rural areas, rural medical institutions might wish to recruit these medical graduates in greater numbers.

Some limitations in this study need to be acknowledged. First, there might be a positive selection bias towards medical students’ intentions to choose rural medical work. Moreover, intention did not always equate to future actual behavior and career choice, and it was unlikely that all medical students who reported intending to work in rural medical institutions after graduation would actually do so, in addition to some students who reported intending to work in urban areas actually spending some time working in rural locations. Second, the rural clinical clerkship was not compulsory for medical students, and it appeared that the medical students who had experience with a rural clinical clerkship were interested in rural medical work and voluntarily chose a rural medical institution for a clinical clerkship. Thus, the association between a rural clinical clerkship and intention of rural medical work in this study could not be considered to be definitive, as medical students’ intentions might be attributed to their personal interest in rural medical work even without the experience of a rural clinical clerkship. Third, we did not analyze the impact of the duration of a rural clinical clerkship in this study; however, it was a critical element, since there might be vast differences between medical students undergoing a year in rural placement and those with only a few days of rural medical experience with regard to their intentions to choose rural medical work. Fourth, many potential influencing factors identified in previous studies were not analyzed in this study due to limited variables set in the questionnaire. Fifth, since the study was of a cross-sectional type, the association observed in the study could not be assumed to represent a causal relation.

## Conclusions

This study demonstrated a significant association between rural clinical clerkships and medical students’ intentions to work in rural medical institutions after graduation in western China. In medical students who had experience of rural clinical clerkship, a 1.63-fold increase was seen in the odds of having intentions to engage in rural medical work. This remained significant but reduced to a 1.24-fold increase when all other possible predictors were also taken into account. For the medical students with a rural background, a rural clinical clerkship did not have significant impact on their intentions to choose rural medical work in a multivariate logistic regression; for students with an urban background, a rural clinical clerkship increased the odds of having intentions to choose rural medical work by 2.10-fold. Related policy makers might consider developing compulsory rural clerkship programs and implementing them among medical students to increase early rural exposure.

## Supporting information

S1 File Questionnaire. (PDF)

S2 File Data set. (SAV)

## References

[1] Shamian J. No health without a workforce, no workforce without nurses. British Journal of Nursing. 2016;25: 54 doi: 10.12968/bjon.2016.25.1.54 26768046 10.12968/bjon.2016.25.1.54

[2] National Bureau of Statistics of China. China statistical yearbook 2016 [Internet]. Beijing: China Statistics Press; 2016 http://www.stats.gov.cn/tjsj/ndsj/2016/indexeh.htm

[3] National Health and Family Planning Commission of the People’s Republic of China. China health and family planning statistical yearbook 2015. Beijing: Peking Union Medical College Press; 2015.

[4] Budhathoki SS, Zwanikken PA, Pokharel PK, Scherpbier AJ. Factors influencing medical students’ motivation to practise in rural areas in low-income and middle-income countries: a systematic review. BMJ open. 2017;7: e013501 doi: 10.1136/bmjopen-2016-013501 28232465 10.1136/bmjopen-2016-013501 PMC5337703

[5] Huntington I, Shrestha S, Reich NG, Hagopian A. Career intentions of medical students in the setting of Nepal’s rapidly expanding private medical education system. Health Policy and Planning. 2012;27: 417–28. doi: 10.1093/heapol/czr052 21880690 10.1093/heapol/czr052

[6] Ahmed SM, Majumdar MA, Karim R, Rahman S, Rahman N. Career choices among medical students in Bangladesh. Advances in Medical Education & Practice. 2010;2: 51. 10.2147/AMEP.S13451 PMC3661246 23745076

[7] Burch VC, Mckinley D, Van WJ, Kiguli-Walube S, Cameron D, Cilliers FJ, et al Career intentions of medical students trained in six sub-Saharan African countries. Education for Health. 2011;24: 614 22267357

[8] Deressa W, Azazh A. Attitudes of undergraduate medical students of Addis Ababa University towards medical practice and migration, Ethiopia. BMC Medical Education. 2012;12: 68–68. doi: 10.1186/1472-6920-12-68 22867022 10.1186/1472-6920-12-68 PMC3492144

[9] Wyk JMV, Naidoo SS, Esterhuizen TM. Will graduating medical students prefer to practise in rural communities? Official Journal of the South African Academy of Family Practice/primary Care. 2010;52: 149–153.

[10] Diwan V, Minj C, Chhari N, Costa AD. Indian medical students in public and private sector medical schools: are motivations and career aspirations different?–studies from Madhya Pradesh, India. BMC Medical Education. 2013;13: 127 doi: 10.1186/1472-6920-13-127 24034988 10.1186/1472-6920-13-127 PMC3851318

[11] Chuenkongkaew WL, Negandhi H, Lumbiganon P, Wang W, Mahmud K, Cuong PV. Attitude towards working in rural area and self-assessment of competencies in last year medical students: A survey of five countries in Asia. BMC Medical Education. 2016;16 doi: 10.1186/s12909-016-0719-9 27604389 10.1186/s12909-016-0719-9 PMC5015323

[12] Puddey IB, Playford DE, Mercer A. Impact of medical student origins on the likelihood of ultimately practicing in areas of low vs high socio-economic status. BMC Medical Education. 2017;17 doi: 10.1186/s12909-016-0842-7 28056975 10.1186/s12909-016-0842-7 PMC5215143

[13] Playford D, Ngo H, Gupta S, Puddey IB. Opting for rural practice: the influence of medical student origin, intention and immersion experience. The Medical Journal of Australia. 2017;207: 154–158. doi: 10.5694/mja16.01322 28814216 10.5694/mja16.01322

[14] Qing Y, Hu G, Chen Q, Peng H, Li K, Wei J, et al Factors that influence the choice to work in rural township health centers among 4,669 clinical medical students from five medical universities in Guangxi, China. Journal of Educational Evaluation for Health Professions. 2015;12: 40 doi: 10.3352/jeehp.2015.12.40 26268830 10.3352/jeehp.2015.12.40 PMC4536338

[15] Bowman RC, Grouse BJ. Community-driven Medical Education: The Rural Component. The Journal of Rural Health. 2003;19: 214–217. 12839127 10.1111/j.1748-0361.2003.tb00564.x

[16] Organization WH. Increasing access to health workers in remote and rural areas through improved retention: global policy recommendations. World Health Organization; 2010. 23741785

[17] Barrett FA, Lipsky MS, Nawal Lutfiyya M. The Impact of Rural Training Experiences on Medical Students: A Critical Review. Academic Medicine. 2011;86: 259–263. doi: 10.1097/ACM.0b013e3182046387 21169781 10.1097/ACM.0b013e3182046387

[18] Ishimaru N, Takayashiki A, Maeno T, Kawamura Y, Kurihara H, Maeno T. The impact of an early_exposure program on medical students’ interest in and knowledge of rural medical practices: a questionnaire survey. Asia Pacific Family Medicine. 2015;14 doi: 10.1186/s12930-015-0021-8 25883530 10.1186/s12930-015-0021-8 PMC4399156

[19] Roberts C, Daly M, Kumar K, Perkins D, Richards D, Garne D. A longitudinal integrated placement and medical students’ intentions to practise rurally: Rural integrated placements and medical career intentions. Medical Education. 2012;46: 179–191. 22239332 10.1111/j.1365-2923.2011.04102.x

[20] Halaas GW, Zink T, Finstad D, Bolin K, Center B. Recruitment and retention of rural physicians: outcomes from the rural physician associate program of Minnesota. The Journal of Rural Health. 2008;24: 345–352. doi: 10.1111/j.1748-0361.2008.00180.x 19007388 10.1111/j.1748-0361.2008.00180.x

[21] Smucny J, Beatty P, Grant W, Dennison T, Wolff LT. An evaluation of the rural medical education program of the State University of New York Upstate Medical University, 1990–2003. Academic Medicine. 2005;80: 733–738. 16043527 10.1097/00001888-200508000-00006

[22] Rabinowitz HK, Diamond JJ, Markham FW, Rabinowitz C. Long-term retention of graduates from a program to increase the supply of rural family physicians. Academic Medicine. 2005;80: 728–732. 16043525 10.1097/00001888-200508000-00004

[23] Capstick S, Beresford R, Gray A. Rural pharmacy in New Zealand: Effects of a compulsory externship on student perspectives and implications for workforce shortage. Australian Journal of Rural Health. 2008;16: 150–155. doi: 10.1111/j.1440-1584.2008.00965.x 18471185 10.1111/j.1440-1584.2008.00965.x

[24] Borracci RA, Arribalzaga EB, Couto JL, Dvorkin M, Ahuad Guerrero RA, Fernandez C, et al Factors affecting willingness to practice medicine in underserved areas: a survey of Argentine medical students. Rural and remote health. 2015;15 http://www.rrh.org.au/articles/subviewlatinamer.asp?ArticleID=3485 26625931

[25] Jones MP, Bushnell JA, Humphreys JS. Are rural placements positively associated with rural intentions in medical graduates? Medical Education. 2014;48: 405–416. doi: 10.1111/medu.12399 24606624 10.1111/medu.12399

[26] Somers GT, Spencer RJ. Nature or nurture: The effect of undergraduate rural clinical rotations on pre-existent rural career choice likelihood as measured by the SOMERS Index: NATURE, NURTURE AND RURAL CAREER CHOICE. Australian Journal of Rural Health. 2012;20: 80–87. 22435768 10.1111/j.1440-1584.2012.01258.x

[27] Wilkinson D, Laven G, Pratt N, Beilby J. Impact of undergraduate and postgraduate rural training, and medical school entry criteria on rural practice among Australian general practitioners: national study of 2414 doctors. Medical education. 2003;37: 809–814. 12950945 10.1046/j.1365-2923.2003.01596.x

[28] Zhang L, Bossert T, Mahal A, Hu G, Guo Q, Liu Y. Attitudes towards primary care career in community health centers among medical students in China. BMC Family Practice. 2016;17 doi: 10.1186/s12875-016-0472-5 27423474 10.1186/s12875-016-0472-5 PMC4947335

[29] Arscott-Mills T, Kebaabetswe P, Tawana G, Mbuka DO, Makgabana-Dintwa O, Sebina K, et al Rural exposure during medical education and student preference for future practice location—a case of Botswana. African Journal of Primary Health Care & Family Medicine. 2016;8 doi: 10.4102/phcfm.v8i1.1039 27380783 10.4102/phcfm.v8i1.1039 PMC4926713

[30] Kirschbaum M, Khalil H, Talyor S, Page AT. Pharmacy students’ rural career intentions: Perspectives on rural background and placements. Currents in Pharmacy Teaching and Learning. 2016;8: 615–621. doi: 10.1016/j.cptl.2016.06.002

[31] Playford DE, Evans SF, Atkinson DN, Auret KA, Riley GJ. Impact of the Rural Clinical School of Western Australia on work location of medical graduates. Med J Aust. 2014;200: 104–107. 24484114 10.5694/mja13.11082

[32] Sapkota BP, Amatya A. What factors influence the choice of urban or rural location for future practice of Nepalese medical students? A cross-sectional descriptive study. Human Resources for Health. 2015;13 doi: 10.1186/s12960-015-0084-5 26556580 10.1186/s12960-015-0084-5 PMC4640225

[33] Grobler L, Marais BJ, Mabunda SA, Marindi PN, Reuter H, Volmink J. Interventions for increasing the proportion of health professionals practising in rural and other underserved areas. Cochrane Database of Systematic Reviews. 2009;1: CD005314. 10.1002/14651858.CD005314.pub2 19160251

[34] Bailey N, Mandeville KL, Rhodes T, Mipando M, Muula AS. Postgraduate career intentions of medical students and recent graduates in Malawi: a qualitative interview study. BMC medical education. 2012;12: 87 doi: 10.1186/1472-6920-12-87 22978475 10.1186/1472-6920-12-87 PMC3480922

[35] Andrew R, Gavin MG, Laura C. Review of the Umthombo Youth Development Foundation scholarship scheme, 1999–2013: Afr J Prim Health Care Fam Med. 2015;7: 1–6. 10.4102/phcfm.v7i1.739 PMC4866613 26245594

[36] Winn CS, Chisholm BA, Hummelbrunner JA. Factors affecting recruitment and retention of rehabilitation professionals in Northern Ontario, Canada: a cross-sectional study. Rural & Remote Health. 2014;14: 2619. 24717094

[37] Sen GT, Woolley T, Murray R, Hays R, Mccloskey T. Positive impacts on rural and regional workforce from the first seven cohorts of James Cook University medical graduates. Rural & Remote Health. 2014;14: 2657. 24645878

[38] Li X, Liu S. Status Analysis and Consideration of Medical Education System in China and Abroad. Higher Education of Social Science. 2012;3.

[39] Van Wyk J, Naidoo S, Esterhuizen T. Will graduating medical students prefer to practise in rural communities? South African Family Practice. 2010;52: 149–153. doi: 10.1080/20786204.2010.10873958

