# Supplementary material for: Association between rural clinical clerkship and medical students’ intentions to choose rural medical work after graduation: A cross-sectional study in western China
Source: PLoS One. 2018 Apr 2;13(4):e0195266. doi: 10.1371/journal.pone.0195266 (PMC5880380; doi:10.1371/journal.pone.0195266)
Supplement: S1 File — (PDF) [file pone.0195266.s001.pdf]

Questionnaire for medical students related to this study

(part of the completed questionnaire)

Name of medical school: ____________________________________

Address: ____

Instruction: Please choose one selection only.

No.	Question	Reply
1	Gender<br>(1) Female; (2) Male	
2	Age: _____years	
3	Residence<br>(1) Rural; (2) Urban	
4	Family monthly income per capita<br>(1) ≤ 499 Yuan; (2) 500-999 Yuan; (3) 1000-1999 Yuan;<br>(4) 2000-4999 Yuan; (5) 5000-9999 Yuan; (6) ≥ 10000 Yuan	
5	Education of father<br>(1) Illiteracy; (2) Primary school; (3) Junior high school;<br>(4) Senior high school; (5) Junior college or above	
6	Education of mother<br>(1) Illiteracy; (2) Primary school; (3) Junior high school;<br>(4) Senior high school; (5) Junior college or above	
7	Grade<br>(1) Third year; (2) Fourth year; (3) Fifth year	
8	Specialty<br>(1) General practice; (2) Clinical medicine; (3) Public health; (4) Other	
9	Do you have the experience of clinical clerkship?<br>(1) Yes; (2) No	
10	If yes, where do you practice?<br>(1) Urban; (2) Rural; (3) Other___	
11	Are you willing to work in rural medical institutions after graduation?<br>(1) No; (2) Yes	

CMB 项目医学院校学生调查问卷（部分题项）

院校名称：____

院校地址：________省（自治区）_________市__________区（县）____________

序号	问题及选项	回答
1	性别：<br>(1)女 (2)男	
2	年龄：_____岁	
3	户籍所在地：<br>(1)农村 (2)城镇	
4	您全家的人均月收入是多少？<br>(1)500 元以内 (2)500-999 元 (3)1000-1999 元<br>(4)2000-4999 元 (5)5000-9999 元 (6)10000 元以上	
5	您父亲的文化程度<br>(1)文盲（半文盲） (2)小学 (3)初中 (4)高中/中专 (5)大专及以上	
6	您母亲的文化程度<br>(1)文盲（半文盲） (2)小学 (3)初中 (4)高中/中专 (5)大专及以上	
7	年级：<br>(1)大三 (2)大四 (3)大五	
8	所学专业：<br>(1)全科医学 (2)临床医学 (3)公共卫生 (4)其它	
9	您有过医学实习的经历吗？<br>(1)有 (2)没有	
10	如有，您实习所在的医疗卫生机构所在地是？<br>(1)城市 (2)农村 (3)其他___	
11	您是否愿意毕业后去农村医疗卫生机构（县级医院、乡镇卫生院）工作？<br>(1)不愿意 (2)愿意	
